# Unilateral and bilateral load-velocity relationships in athletes: evidence from a study in boxers

**DOI:** 10.3389/fspor.2025.1598396

**Published:** 2025-10-27

**Authors:** Yemin Han, Yiqing Xie, Zhen Niu, Jiawang Jia, Zhen Zhang

**Affiliations:** ^1^ School of Physical Education, Shanghai University of Sport, Shanghai, China; ^2^ School of Athletic Performance, Shanghai University of Sport, Shanghai, China

**Keywords:** combat sports, strength training, velocity-based resistance training, load-velocity relationship, unilateral and bilateral exercises

## Abstract

**Objective:**

This study aimed to investigate the load-velocity (L-V) relationship in boxers during unilateral (Bulgarian split-squat, BSS) and bilateral (back-squat, BS) lower-limb exercises, and to compare the mean velocity (MV) outputs between these two modalities, with the ultimate goal of providing an evidence-based foundation for optimizing strength training in boxing.

**Methods:**

Twenty trained boxers (age: 19.7 ± 1.0 years) performed incremental loading tests on a Smith machine equipped with a linear position transducer (GymAware) to record MV. Unilateral testing was performed on each leg in a randomized order, with loading progressed incrementally from 30% to 100% of the predicted one repetition maximum (1RM). Participants performed three repetitions at 30%–70% predicted 1RM, two repetitions at 75%–90% predicted 1RM, and one repetition at 95%–100% predicted 1RM, with a 10-second rest between repetitions and a 5-minute rest between load conditions.

**Results:**

We found a close relationship between MV and relative load (%1RM) in both BSS and BS exercises for the non-dominant legs (coefficient of determination; *R*^2^ = 0.94, standard error of estimate; SEE = 0.05 m·s^−1^), dominant legs (*R*^2^ = 0.94, SEE = 0.05 m·s^−1^), and back-squat (*R*^2^ = 0.95, SEE = 0.05 m·s^−1^), reflecting a nearly perfect relationship as per standard interpretations of coefficient strength. Compared to Bulgarian split-squat, back-squat exhibited significantly higher MV at the same %1RM (*P* < 0.01, *η*² = 0.256).

**Conclusion:**

This study validated the use of velocity-based resistance training (VBT) to optimize strength training in boxing. Both unilateral and bilateral exercises showed consistent L-V relationships, supporting individualized load prescription. Bilateral exercises enhanced velocity output, while unilateral exercises helped correct inter-limb strength asymmetries and improve sport-specific stability.

## 1 Introduction

Boxing is a high-intensity sport that demands athletes to generate maximal strength within an extremely short time frame to execute effective punches and footwork (1, 2). The primary source of strength in boxing originates from the lower limbs (3–5), particularly during rapid changes of direction and explosive movements (6). Previous research has demonstrated that lower-limb strength not only contributes directly to movement execution but also plays a pivotal role in transmitting force to the upper body, thereby producing more powerful punches (6–8). Accordingly, lower-limb strength training constitutes a critical component of performance enhancement in boxing (7, 9).

Currently, squat-based exercises are widely employed to enhance lower-limb strength in boxers (3, 6). Among these, bilateral exercises such as the back-squat (BS) are more commonly practiced, as they effectively improve overall strength and stability, activate large muscle groups, and generate greater absolute strength outputs (10, 11). Despite these advantages, bilateral training is often associated with the phenomenon of the “bilateral deficit”, where the combined strength produced by both limbs simultaneously is less than the sum of their respective unilateral outputs. In contrast, unilateral exercises represented by the Bulgarian split-squat (BSS) align more closely with the sport-specific demands of boxing (12). BSS replicates the asymmetric strength production inherent in punching and footwork movements (6, 13), enhances single-leg strength performance, and helps to reduce inter-limb strength imbalances (13, 14). Recent studies have confirmed the effectiveness of both unilateral and bilateral resistance training in improving lower-limb strength and punching performance in boxers. For example, Liu et al. (5) demonstrated that combined unilateral and bilateral training, which included BS and single-leg exercises, significantly enhanced squat and bench press strength in adolescent boxers. Similarly, Liu et al. (15) reported that variable resistance training within a complex protocol led to notable gains in strength and punching power in elite amateur boxers. These findings support the inclusion of both squat variations in boxing-specific strength programs, highlighting their complementary roles in developing maximal strength and sport-specific performance.

In boxing, most actions (e.g., punching and defensive maneuvers) not only require single-leg support and unilateral strength production but also demand that these movements be executed at maximal velocity (3, 14). Consequently, integrating movement velocity into strength training is critical for boxing performance. In recent years, velocity-based resistance training (VBT) has emerged as a precise method for guiding resistance training. It is grounded in the load-velocity (L-V) relationship, whereby movement velocity systematically decreases as relative load (%1RM) increases (16, 17). VBT enables real-time monitoring of barbell velocity to dynamically adjust training loads, allowing for targeted development across specific velocity zones. This is particularly valuable in boxing, where rapid strength production is essential (3). Although extensive research has validated the reliability of the L-V relationship in bilateral exercises such as squats and deadlifts (18, 19), its application in unilateral training remains underexplored—especially considering the added challenges of stability and inter-limb asymmetries inherent to single-leg movements (14, 20). Additionally, most VBT protocols estimate training loads using grouped L-V profiles (GLVP), which may neglect individual variability among athletes. Recent studies have highlighted the advantages of individualized L-V profiling (ILVP), which incorporates athlete-specific characteristics and may improve the accuracy of VBT implementation (21, 22). Therefore, examining the differences between ILVP and GLVP in both unilateral and bilateral training contexts is particularly important in combat sports, where inter-limb asymmetry is prevalent.

The aim of the study was not only to identify performance differences between exercise modalities, but also to provide insight into kinetic chain functionality and force transmission in boxing-relevant movements. Understanding how load and velocity interact across unilateral and bilateral patterns contributes to optimizing neuromuscular recruitment and enhancing overall striking performance. Specifically, the objectives were: (1) to compare mean velocity (MV) outputs between unilateral and bilateral exercises across various %1RM loads; (2) to examine potential differences in the L-V relationship between the dominant and non-dominant legs during unilateral exercises, thereby assessing strength symmetry; (3) to evaluate the accuracy and applicability of ILVP compared to GLVP; and (4) to provide practical guidance for optimizing VBT protocols in boxing. We formulated three exercise-specific hypotheses: First, MV would be significantly higher in bilateral exercises than in unilateral exercises at equivalent %1RM, due to greater stability and muscle recruitment (10). Second, despite possible unilateral strength asymmetries during BSS exercise, we expected no significant difference in the L-V curve patterns between the dominant and non-dominant legs, reflecting symmetrical neuromuscular control (13). Third, we hypothesized that ILVP would show higher predictive accuracy and lower estimation error than GLVP, enabling more precise and individualized load prescriptions for strength training in boxers (23, 24). Clarifying these relationships will help coaches and rehabilitation professionals better tailor training loads, differentiate between systemic and limb-specific performance factors, and safely implement individualized VBT strategies in combat sports.

## 2 Materials & methods

### 2.1 Experimental design

This study used a cross-sectional research design to analyze the relationship between movement speed and %1RM in the exercises. We selected two common lower limb training movements, the back-squat (BS) and Bulgarian split-squat (BSS). All tests were conducted at the Physical Training Research Center of Shanghai University of Sport (Shanghai, China). Participants visited the laboratory for five sessions, with a 72 h interval between each test to minimize the effects of fatigue. The study protocol included one familiarization session and four testing sessions. In session 1, the researchers explained the experimental protocol and familiarized the participants with the test equipment. Then, 1RM assessments of the BS and BSS were conducted in sessions 2 and 3, and finally, MV for every %1RM loads was measured in sessions 4 and 5 based on the 1RM values obtained in the previous two sessions. In the familiarization session, the dominant leg of each participant was determined by asking which leg they primarily use when kicking, with the opposite leg designated as the non-dominant leg (25). After determining the dominant leg, participants performed moderate-intensity BSS and BS exercises on a Smith machine (Lipper, Nantong, China) to ensure proper technique and maximal execution speed.

### 2.2 Subjects

The sample size was calculated using G*Power 3.1 (26) based on a repeated-measures ANOVA (within-subjects design) with one group and three measurement conditions (i.e., back-squat, dominant-leg BSS, and non-dominant-leg BSS). The statistical parameters were set as follows: moderate effect size (f = 0.25), significance level (*α* = 0.05), and desired statistical power (1-*β*) = 0.80. The power analysis indicated that a minimum of 18 participants would be required to detect medium effects with sufficient statistical power under this within-subjects design. A total of 20 university-level boxers (see Table 1) from Shanghai University of Sport (Shanghai, China) voluntarily participated. Participants were familiarized with the research protocol and provided written informed consent after receiving information about potential risks and benefits. Inclusion criteria ensured valid data collection and minimized injury risks: 1) no injuries in the past 6 months, 2) no additional training outside the study during the test period, and 3) at least 2 years of strength training experience (2–4 sessions per week). The study was approved by the Ethics Committee of Shanghai University of Sport (Approval No. 102772024RT050).

**Table 1:** Baseline characteristics of study participants.

Characteristic	*n* = 20
Age (years)	19.65 ± 0.99
Body mass (kg)	77.90 ± 8.48
Height (cm)	180.85 ± 6.62
Boxing experience (years)	5.95 ± 1.36
Back-squat 1 RM (kg)	135.25 ± 30.21
Bulgarian split-squat 1 RM (kg)	
Dominant leg	95.25 ± 25.36
Non-dominant leg	90.25 ± 24.89

1RM, one-repetition maximum; Data are expressed as mean ± standard deviation (*n* = 20).

### 2.3 Procedures

#### 2.3.1 One repetition maximum (1RM) assessment

The 1RM test was conducted using both the back-squat (BS) and Bulgarian split-squat (BSS) exercises, adhering to the guidelines of the National Strength and Conditioning Association (NSCA) (27). Prior to testing, participants completed a standardized warm-up consisting of 5 min of cycling at a self-selected pace, followed by 5 min of static stretching and lower-limb joint mobility exercises (targeting the hip, knee, and ankle joints) to elevate heart rate and reduce injury risk (28). Participants then performed 2–3 sets of light-load exercises (e.g., empty barbell) for both BS and BSS with 6–8 repetitions per set to improve movement familiarity and activate relevant musculature. After a 2 min rest, they proceeded to heavier loads. The test began with an initial load of 20 kg and progressed in 10 kg increments. Once the mean velocity (MV) dropped below 0.50 m·s^−1^, the increments were reduced to 1–5 kg. Repetition volume was regulated based on the measured MV: light loads (MV > 0.70 m·s^−1^) were performed for 3–4 repetitions, moderate loads (0.50–0.70 m·s^−1^) for 2 repetitions, and heavy loads (MV < 0.50 m·s^−1^) for a single repetition. The test was terminated when participants were unable to complete the lift with proper technique or when barbell MV fell below 0.20 m·s^−1^, indicating maximal effort (17). Rest periods were standardized: 2–3 min between light and moderate loads and 3–5 min between heavy load attempts.

**Figure 1:**
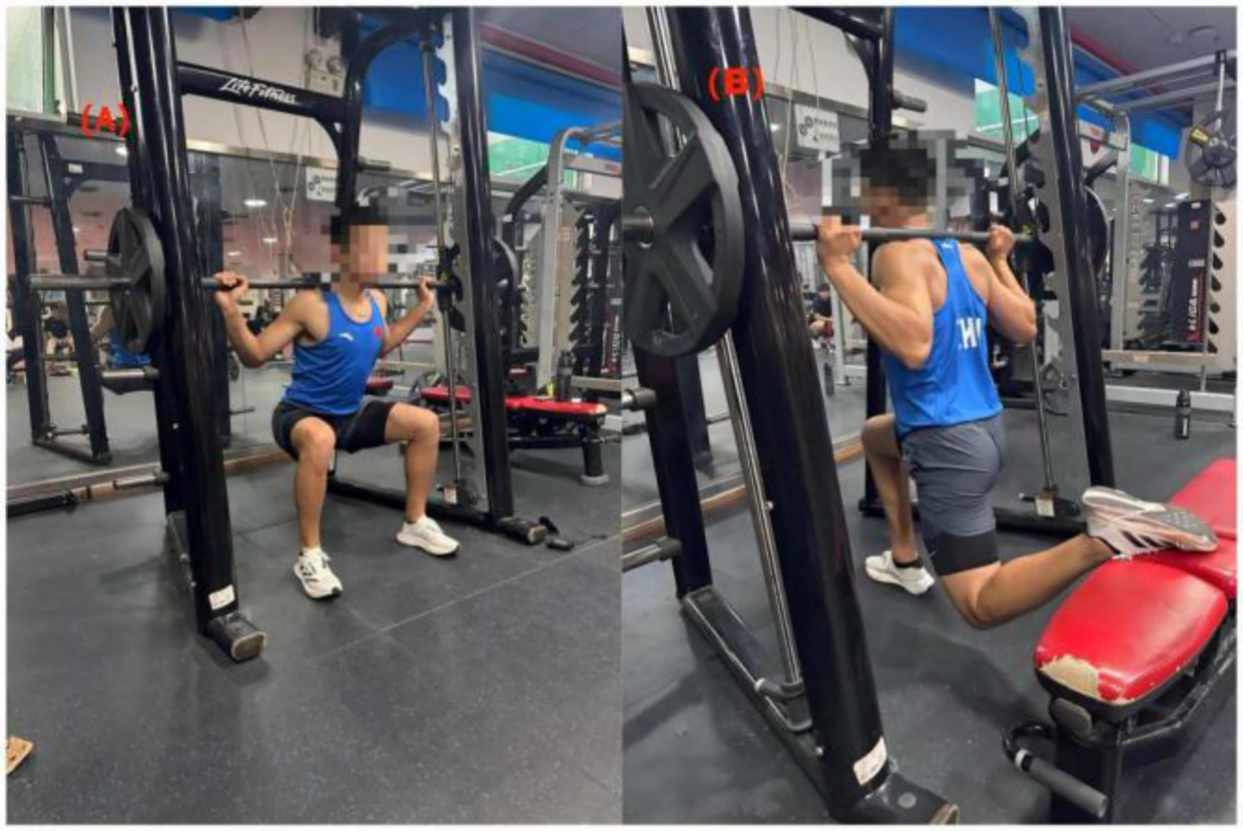
The initial set-up of **(A)** back-squat and **(B)** Bulgarian split-squat exercises.

For the BS, the high-bar back-squat technique was used (29). Participants positioned their feet shoulder-width apart or slightly wider, with toes pointing forward. The knees were aligned with the direction of the toes throughout the movement. During the descent, participants allowed their knees to pass slightly beyond their toes to minimize shear forces on the spine. The squat depth was standardized such that the thighs were parallel to the ground, with knee angles ranging between 60° and 70°, as shown in Figure 1A.

For the BSS, participants were instructed to elevate their rear foot on a bench while keeping the front foot flat on the ground and maintaining an upright torso. To standardize the elevation of the non-weight-bearing leg, the rear foot was positioned on an adjustable-height bench. This ensured that the front thigh reached parallel to the ground with the front knee flexed to approximately 90°, thereby accommodating individual differences in leg length and participant height. The exercise commenced from a standing position, followed by a controlled descent until the front thigh was parallel to the ground and the rear knee approached (but did not touch) the floor. During the descent, the front knee was flexed to approximately 90°, while the rear hip maintained a neutral alignment around 180°, as illustrated in Figure 1B (30).

#### 2.3.2 Mean velocity (MV) test

Before testing, the linear position transducer (GymAware Power Tool Version 6.1; Canberra, Australia) was properly installed and connected to the barbell of the Smith machine to measure instantaneous velocity. The device sampled at a rate of >50 Hz to ensure reliable data collection throughout the entire range of motion. In the MV testing, we employed incremental loads of 30%, 35%, 40%, 45%, 50%, 55%, 60%, 65%, 70%, 75%, 80%, 85%, 90%, 95%, and 100% of predicted 1RM. This load selection was based on previous studies (18, 31) and refined through pilot testing to ensure feasibility and data reliability. Smaller increments were chosen compared to using 10% 1RM to precisely capture MV variations across different load levels and reduce measurement errors. To isolate the concentric phase and minimize stretch-shortening cycle (SSC) effects, participants were instructed to pause briefly (3–4 s) at the bottom position before initiating the upward movement, ensuring pure concentric effort (pause method).

In the BSS test, the left and right legs were tested separately in a random order. Participants performed three repetitions at 30%–70% of predicted 1RM, two repetitions at 75%–90% of predicted 1RM, and one repetition at 95%–100% of predicted 1RM, with a 10 s rest between repetitions and a 5 min rest between different load conditions MV was recorded for each repetition, and the highest value from each set was analyzed. During each repetition, strong verbal encouragement and velocity feedback were provided to motivate participants to exert maximal effort.

### 2.4 Statistical analyses

Descriptive data were presented as means and standard deviations (SD). The L-V relationship was adjusted using second-order polynomial regression, as this model provided the best fit (32, 33). The relationship between MV and %1RM was evaluated using the coefficient of determination (*R*^2^), standard error of estimate (SEE), 95% confidence intervals (CI), and the coefficient of variation (CV = SD/mean × 100) to assess model accuracy and predictive reliability. Before conducting the repeated-measures ANOVA test, we performed the Shapiro–Wilk test to assess the normality of the data and Mauchly's test of sphericity to evaluate homogeneity of variance assumptions. When violations of sphericity were detected, Greenhouse-Geisser corrections were applied to adjust the degrees of freedom (34). Additionally, we conducted Tukey HSD *post-hoc* tests for multiple comparisons and reported effect sizes (*η*^2^) to quantify the magnitude of differences between conditions. Interpretations of effect size were evaluated (35) at the following levels: small effect (0.01–0.058), medium effect (0.059–0.137), and large effect (>0.138). These measures ensured the robustness and reliability of our statistical analyses. Statistical significance was set at *p* ≤ 0.05, and all analyses were performed using SPSS (version 27, IBM, Armonk, NY, USA).

## 3 Results

The mean 1RM for the BS was 135.3 ± 30.2 kg, while the mean 1RM for the dominant and non-dominant legs in the BSS were 95.3 ± 25.4 kg and 90.3 ± 24.9 kg, respectively. Table 2 presents the mean MV values and standard deviations for the BS exercise at various loads, along with those for the dominant and non-dominant legs in the BSS exercise. The comparison of MV between BS and BSS exercises was based on the average MV of the dominant and non-dominant legs in BSS exercise. At lower loads (30%–60% 1RM), the non-dominant legs demonstrated slightly higher MV than the dominant legs, whereas at higher loads (70%–100% 1RM), their MV values converged. MV was significantly higher in bilateral exercise across all loads compared to unilateral exercise, suggesting an advantage in speed and stability. Since MV values for the dominant and non-dominant legs did not differ significantly (*p* > 0.05) at corresponding %1RM loads, their data were merged into a single predictive equation.

**Table 2:** Mean velocity for every percentage of one-repetition maximum in back-squat and Bulgarian split-squat exercises.

%1RM	Back-squat MV (m·s^−1^)	Bulgarian split-squat MV (m·s^−1^) dominant legs non-dominant legs		*p*	*η* ^2^
30	1.12 ± 0.08	0.91 ± 0.07	0.93 ± 0.08	<0.001	0.87
35	1.06 ± 0.07	0.88 ± 0.07	0.88 ± 0.08	<0.001	0.76
40	1.00 ± 0.07	0.84 ± 0.07	0.84 ± 0.07	<0.001	0.69
45	0.94 ± 0.07	0.80 ± 0.06	0.80 ± 0.07	<0.001	0.68
50	0.88 ± 0.06	0.74 ± 0.06	0.74 ± 0.06	<0.001	0.71
55	0.81 ± 0.06	0.68 ± 0.05	0.68 ± 0.06	<0.001	0.81
60	0.78 ± 0.05	0.62 ± 0.05	0.63 ± 0.06	<0.001	0.85
65	0.71 ± 0.05	0.58 ± 0.05	0.59 ± 0.05	<0.001	0.76
70	0.66 ± 0.05	0.53 ± 0.05	0.54 ± 0.05	<0.001	0.78
75	0.60 ± 0.05	0.48 ± 0.04	0.49 ± 0.04	<0.001	0.82
80	0.55 ± 0.04	0.44 ± 0.04	0.45 ± 0.04	<0.001	0.83
85	0.49 ± 0.04	0.39 ± 0.03	0.39 ± 0.03	<0.001	0.79
90	0.44 ± 0.03	0.35 ± 0.02	0.35 ± 0.02	<0.001	0.85
95	0.38 ± 0.03	0.31 ± 0.02	0.30 ± 0.02	<0.001	0.85
100	0.31 ± 0.03	0.25 ± 0.02	0.25 ± 0.02	<0.001	0.67

%1RM, percentage of one-repetition maximum; MV, mean velocity; Data are expressed as mean ± standard deviation (*n* = 20).

**Figure 2:**
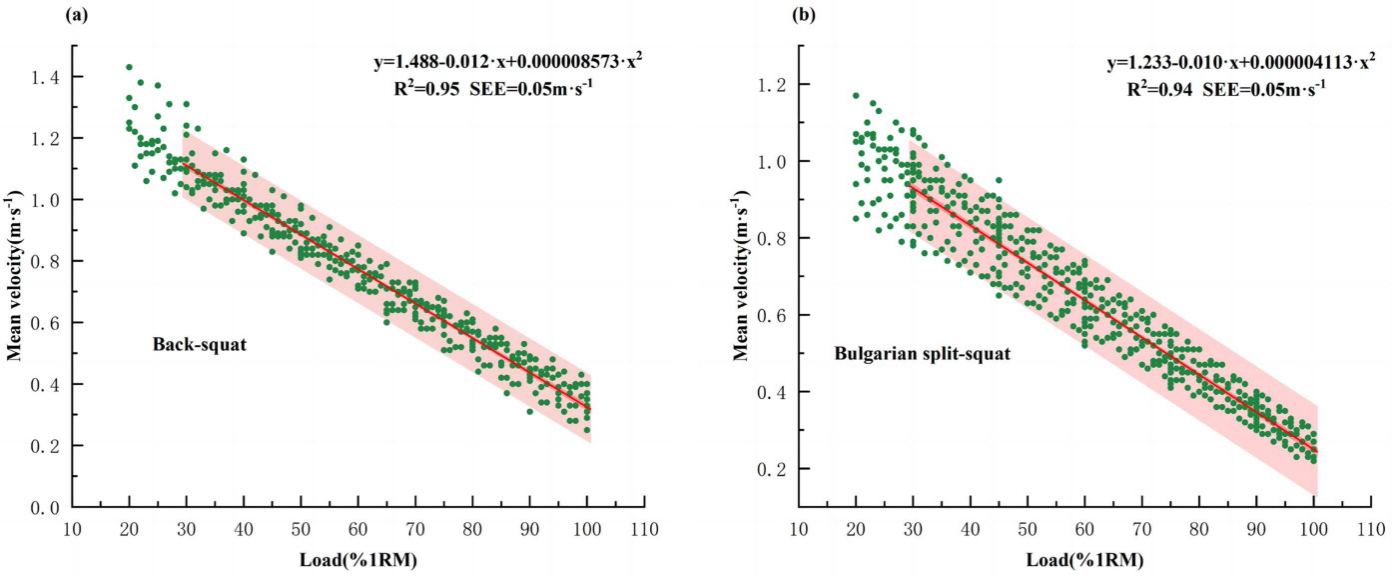
Relationship between relative load (%1RM) and MV in the (a) back-squat and (b) Bulgarian split-squat exercises. Second-degree polynomial fit; MV, mean velocity; *R*^2^, coefficient of determination; SEE, standard error of estimation.

As shown in Figure 2, a second-order polynomial regression analysis was conducted to model the L-V relationship for the unilateral and bilateral exercises. Results showed a close polynomial relationship for all conditions: dominant legs (*R*^2^ = 0.94, SEE = 0.04 m·s^−1^), non-dominant legs (*R*^2^ = 0.94, SEE = 0.05 m·s^−1^), and back-squat (*R*^2^ = 0.95, SEE = 0.05 m·s^−1^), with low prediction error (SEE = 0.05 m·s^−1^). Based on the interpretative scale proposed by Hopkins (36), all observed *R*^2^ values reflect nearly perfect strength of association. Compared to the grouped load-velocity profile (GLVP), the individualized load-velocity profile (ILVP) provided a better fit in Table 3.

**Table 3:** Validity comparison between ILVP and GLVP in the back-squat and the Bulgarian split-squat exercise.

LVP	*R* ^2^	Strength Classification	SEE (m·s^−1^)	95% CI	CV (%)	*p*
ILVP-BSS	0.99 ± 0.003	Nearly perfect	0.02 ± 0.003	[0.42, 0.80]	0.36	<0.001
GLVP-BSS	0.94	Nearly perfect	0.05	[0.57, 0.61]	0.37	
ILVP-BS	0.99 ± 0.01	Nearly perfect	0.03 ± 0.01	[0.58, 0.86]	0.35	<0.001
GLVP-BS	0.95	Nearly perfect	0.05	[0.69, 0.75]	0.35	

LVP, load velocity profile; ILVP, individual load velocity profile; GLVP, group load velocity profile; *R*^2^, coefficient of determination; SEE, standard error of estimate; CV, coefficient of variability; CI, confidence interval; Data are expressed as mean ± standard deviation (*n* = 20).

The sphericity test showed that the assumption of sphericity for load (%1RM) was violated (W < 0.001, *p* < 0.001). Therefore, the Greenhouse-Geisser correction was applied in the within-subject effects analysis. The corrected repeated-measures ANOVA test revealed a significant main effect of load (%1RM) on MV (*p* < 0.001, *η*² = 0.981), indicating that changes in load significantly influenced movement velocity.

Although the absolute load (%1RM) differed significantly between the dominant legs and non-dominant legs (*p* < 0.01), their L-V relationships were not significantly different (*p* > 0.05). Back-squat exhibited significantly higher MV at corresponding %1RM loads compared to Bulgarian split-squat (*p* < 0.01, *η*^2^ = 0.256). The *post-hoc* Tukey HSD tests indicated no significant difference between the non-dominant and dominant legs (Mean Difference = 0.003 m·s^−1^, SE = 0.014, *p* = 0.972, 95% CI = [−0.031, 0.038). However, bilateral exercise exhibited significantly higher MV than both the non-dominant legs (Mean Difference = 0.127 m·s^−1^, SE = 0.014, *p* < 0.001, 95% CI = [0.093, 0.161) and the dominant legs (Mean Difference = 0.130 m·s^−1^, SE = 0.142, *p* < 0.001, 95% CI = [0.096, 0.164).

## 4 Discussion

The primary objective of this study was to optimize strength training strategies for boxing by analyzing the L-V relationship in unilateral (Bulgarian split-squat, BSS) and bilateral (back-squat, BS) lower-limb exercises. The findings demonstrated that both unilateral and bilateral exercises exhibited a highly linear L-V relationship (*R*^2^ ≥ 0.94), consistent with previous research (17, 37, 38). While MV outputs were lower during unilateral exercises compared to bilateral exercises, no significant differences in MV were observed between the dominant and non-dominant legs (*p* > 0.05), thereby supporting both the first and second hypotheses of this study.

Notably, ILVP demonstrated higher predictive accuracy than GLVP (*R*^2^ = 0.99 vs. 0.94; SEE = 0.02 vs. 0.05), which supports our third hypothesis. While GLVP provides a population-average model, it may fail to capture the velocity profiles of athletes who deviate significantly from the group mean, particularly those with exceptionally fast or slow lifting characteristics. In contrast, ILVP reflects each athlete's specific force-velocity characteristics and fatigue thresholds, thereby enabling more precise 1RM predictions and individualized velocity targets (23, 24, 39). Moreover, inter-individual variability in the L-V relationship can arise from factors such as anthropometric traits (e.g., limb length, body mass distribution), neuromuscular properties (e.g., motor unit recruitment strategies, fiber type composition), and training history (e.g., prior exposure to unilateral vs. bilateral movements) (40–42). These variables may influence how athletes respond to load and velocity, contributing to deviations from the group-level curve captured by GLVP. Additionally, research has shown that movement velocity may decline due to concentric fatigue even when range of motion remains unchanged (21), meaning coaches relying solely on GLVP could misinterpret transient fatigue as performance decline. From a practical standpoint, ILVP allows strength and conditioning professionals to more accurately tailor training loads to each athlete's physiological profile. This is particularly relevant in sports like boxing, where marginal performance differences may determine competitive outcomes.

The BSS exercise has been shown to effectively reduce lower-limb strength imbalances and enhance unilateral stability in athletes (13, 43). It also enables selective activation of stabilizing muscle groups, such as the gluteus maximus and hamstrings (44, 45). In contrast, BS exercises are widely recognized for their capacity to increase maximal strength, power output, and whole-body stability (6, 18). Meanwhile, bilateral exercises produce higher MV at comparable %1RM loads due to enhanced muscular recruitment and postural stability (10, 11, 46). Therefore, integrating unilateral and bilateral resistance training offers complementary benefits, enabling more comprehensive development of strength, power, and sport-specific motor control in boxers (5, 14, 47).

The VBT guided L-V profile is particularly critical in boxing, as it enables precise regulation of training intensity. Evidence suggests that combat sport athletes generally exhibit a characteristic linear L-V relationship, where movement velocity (e.g., punching speed) systematically decreases with increasing external load, in accordance with the classical force-velocity curve (48). By facilitating real-time monitoring and adjustment of training loads, VBT significantly enhances the efficiency of explosive strength development, with optimal adaptations observed when training within 30%–70% of maximal velocity (37). Additionally, research by Cui et al. (49) demonstrated that modulating the velocity loss (VL) threshold in VBT markedly improved punching performance in boxers, particularly in terms of dominant-side punching strength and velocity. Similarly, Huang et al. (50) found that VBT was superior to autoregulatory progressive resistance exercise (APRE) in enhancing explosive power and agility in taekwondo athletes, especially in tasks requiring rapid strength production. Collectively, these findings provide valuable guidance for optimizing strength training strategies in combat sports.

Although this study provides valuable data on the L-V relationship in unilateral and bilateral exercises among trained boxers, certain limitations must be acknowledged. The use of a Smith machine may have attenuated the balance and stabilization demands typically associated with these movements, potentially influencing the MV outcomes. Future research should incorporate free-weight protocols to better assess the functional stability requirements inherent in unilateral and bilateral training modalities.

## 5 Conclusion and practical applications of the study

### 5.1 Conclusion

This study addressed the applied need to optimize strength training in boxing by validating the use of VBT. The findings confirmed that both unilateral and bilateral exercises exhibit strong and consistent L-V relationships, supporting their use for individualized load prescription. Bilateral exercises demonstrated superior velocity output capacity, while unilateral exercises contributed to reducing inter-limb strength asymmetries and enhancing sport-specific stability. Notably, ILVP showed higher predictive accuracy than GLVP, suggesting that ILVP may better accommodate inter-individual variability in neuromuscular characteristics. These insights provide an evidence-based foundation for developing more precise and effective strength training programs tailored to the physical and performance demands of boxing.

### 5.2 Practical applications

The L-V relationships established for the BS and BSS offer a scientific framework for implementing VBT in boxing. Coaches may initially apply GLVP for broad load estimation in team settings or early training phases where individual testing is not feasible. However, ILVP should be prioritized when aiming for maximal precision, especially in elite or highly variable populations. Individualized profiles allow for more accurate 1RM estimation, finer velocity target setting, and better monitoring of neuromuscular fatigue or adaptation. Additionally, unilateral profiling can help identify and address leg strength imbalances, reducing injury risk and improving functional stability. Overall, individualized training prescriptions appear more effective for optimizing load specificity and long-term performance gains in boxing.

## Data Availability

The datasets presented in this study can be found in online repositories. The names of the repository/repositories and accession number(s) can be found in the article/Supplementary Material.
